# Thirty-Seventh Annual Convention, Detroit, September, 1900

**Published:** 1901-01

**Authors:** 


### A. V. M. A.

### THIRTY-SEVENTH ANNUAL CONVENTION, DETROIT, SEPTEMBER, 1900.

(Continued from page 776.)

*State Secretaries' Reports.*

THE resident State Secretaries' reports were more numerous than usual and more complete in covering the work intended to be done by these officials of the Association.

*Arizona.* Dr. Norton reported the general condition of the animals in that State as to the existence of tuberculosis, Texas fever, black-leg, and glanders, the State being quite free from any serious outbreaks. The stringent sanitary laws provided, he reported, had done much to control all the infectious and contagious diseases.

*California.* Dr. Fred. E. Pierce, for this State, reported an unusual freedom of livestock from all diseases. He reported the State Association in process of dissolution and a lack of interest in matters pertaining to the profession throughout the entire State.

*Illinois.* Dr. E. N. Nighbert reported great activity throughout the entire State. The Association had been very active in the work of new legislation, had made much progress, and much was expected from the new law regulating the practice of veterinary science in the State. He reported 665 veterinarians registered,

288 of whom were graduates of colleges. More attention was being paid by the General Assembly in the line of better food inspection and a more earnest enforcement relative to the inspection of dairies and animals used for commercial purposes in the production of foodstuffs. He reported a general freedom from infectious and contagious diseases, a much less number of cases of glanders reported, and a very great improvement in the general care and treatment of animals, all of which he attributed to the higher education of veterinarians and their more general recognition.

*Michigan.* State Veterinarian Dunphy, as resident State Secretary, reported a general freedom from serious outbreaks from any contagious and infectious diseases among horses, cows, and swine. Parasitic diseases among sheep had given rise to numerous losses. The several outbreaks of anthrax reported were seriously questioned by those who had conducted examinations. There had been little work done in the line of legislation beyond the passage of the bill protecting the title of veterinarian and providing for their proper registration, but it was the general consensus of opinion that the bill passed had done but little to strengthen the profession. He referred to the work of the State Association and their pleasure in welcoming the A. V. M. A. to the State. The matter of reimbursing owners of tuberculous cattle and glandered horses was discussed by Drs. Fair, Leech, Reynolds, and Dunphy.

*Minnesota.* Dr. S. H. Ward reported a greater activity in professional matters, commending very highly the work of Drs. Reynolds, Brimhall, and Annand in stamping out and controlling infectious and contagious diseases. He reported the Minnesota veterinarians active and aggressive in their zeal and interest for the success of army legislation. The cities and towns are taking a more active interest in regard to the purity of their milk supply. He made reference to the various infectious and contagious diseases that the State had dealt with during the past year.

(To be continued.)

---

Dr. E. H. Brown, who was associated with the veterinarians of the United States Army at Manila, P. I., has returned from the service owing to ill health incident to the climate and the trying duties imposed upon him in the service in the Philippines. Dr. Brown fully appreciates the need of an Army Veterinary Corps, with rank, to the veterinarian.

